# Effect of Dietary 4-Phenylbuthyric Acid Supplementation on Acute Heat-Stress-Induced Hyperthermia in Broiler Chickens

**DOI:** 10.3390/ani12162056

**Published:** 2022-08-12

**Authors:** Yukako Tokutake, Ryo Takanashi, Motoi Kikusato, Masaaki Toyomizu, Kan Sato

**Affiliations:** Animal Nutrition, Life Sciences, Graduate School of Agricultural Science, Tohoku University, Sendai 980-8572, Japan

**Keywords:** endoplasmic reticulum stress, acute heat stress, broiler, 4-phenylbuthyric acid

## Abstract

**Simple Summary:**

Heat stress (HS) induces endoplasmic reticulum (ER) stress and disrupts the ER and cellular homeostasis. A recent study showed that ER stress was induced in broiler chickens under severe and acute HS; however, it was unclear how the alleviation of ER stress affects the physiological state of broiler chickens. Therefore, this study aimed to investigate the ameliorative effects of an ER stress alleviator, 4-phenylbutyric acid (4-PBA), which is a chemical chaperone that reduces ER stress, on the body temperature response, energy metabolic state, and cellular ER stress in HS-exposed birds. 4-PBA supplementation did not negatively affect the growth rate. In addition, 4-PBA suppressed the HS-induced ER stress response in skeletal muscle. Surprisingly, 4-PBA significantly decreased body temperature elevation in HS birds. The present study showed that the ER stress, alleviated by 4-PBA, might contribute to the induction of heat tolerance in broiler chickens.

**Abstract:**

Hot, humid weather causes heat stress (HS) in broiler chickens, which can lead to high mortality. A recent study found that HS causes endoplasmic reticulum (ER) stress. However, the possible involvement of ER stress in HS-induced physiological alterations in broiler chickens is unclear. This study aimed to evaluate the effect of the dietary supplementation of 4-phenylbutyric acid (4-PBA), an alleviator of ER stress, in acute HS-exposed young broiler chickens. Twenty-eight 14-day-old male broiler chickens (ROSS 308) were divided into two groups and fed either a control diet or a diet containing 4-PBA (5.25 g per kg of diet feed) for 10 days. At 24 days old, each group of chickens was kept in thermoneutral (24 ± 0.5 °C) or acute HS (36 ± 0.5 °C) conditions for 2 h. The results showed that thermoneutral birds supplemented with 4-PBA exhibited no negative effects in terms of broiler body weight gain and tissue weight compared to non-supplemental birds. HS increased body temperature in both the control and 4-PBA groups, but the elevation was significantly lower in the 4-PBA group than in the control group. The plasma non-esterified fatty acid concentration was significantly increased by HS treatment in non-supplemental groups, while the increase was partially attenuated in the 4-PBA group. Moreover, 4-PBA prevented HS-induced gene elevation of the ER stress markers GRP78 and GRP94 in the skeletal muscle. These findings suggest that the 4-PBA effect may be specific to the skeletal muscle in HS-exposed birds and that 4-PBA supplementation attenuated HS-induced muscle ER stress, which could be associated with a supplementation of the body temperature elevation and lipolysis.

## 1. Introduction

Hot, humid weather causes heat stress (HS) in livestock and results in economic losses [1]. Chickens are particularly sensitive to high temperatures because they lack sweat glands, have abundant feathers, and have a high feed intake and metabolic heat production relative to their body size [1]. Hence, heat-related problems, such as heatstroke and hyperthermia, cause high mortality in broilers [2]. Simultaneously, HS affects the physiology and the systemic energy metabolism levels of broiler chickens [3,4]. It is important to understand the physiological changes associated with HS loads in the whole body and individual organs to adapt to HS because the mitigating effects of HS depend on feed regimen and strain [3,4].

High temperatures alter cell physiology [5], such as causing increases in membrane fluidity [6], protein denaturation, and cell death [7]. Similarly, HS disrupts the function of the endoplasmic reticulum (ER), an organelle involved in protein processing and folding in cells [8,9]. The accumulation of immature and defective proteins in the ER lumen leads to the impairment of ER functions and cell death, a process known as ER stress [10]. ER stress can activate the unfolded protein response (UPR) to restore ER homeostasis. Major UPR sensor proteins, such as activating transcription factor 6 (ATF6), inositol-requiring enzyme 1 (IRE1), and PKR-like endoplasmic reticulum kinase (PERK), are activated under ER stress conditions and initiate a series of protein expressions. ATF6 is transported to the Golgi apparatus and cleaved by proteases under ER stress [11,12]. Cleaved ATF6 is the sensor that responds first [13] and acts as a transcription factor that activates ER-resident molecular chaperones, glucose-regulated protein 78 (GRP78) and GRP94, which are the heat shock protein family proteins that upregulate the folding capacity of the ER [14,15,16,17]. IRE1 splices X-box binding protein 1 (XBP1) mRNA via endonuclease activity [17]. Spliced XBP1 (XBP1s) is an activated form and transcription factor that regulates a range of processes, including protein folding, lipid synthesis, and ER-associated degradation [17]. PERK promotes the phosphorylation of eukaryotic initiation factor 2α (EIF2α) to attenuate ER stress by decreasing protein translation [18]. Phosphorylated EIF2α activates activating transcription factor 4 (ATF4) and the C/EBP homologous protein (CHOP) pathway, triggering cellular apoptosis if the repair process fails [19].

A recent investigation reported that both ER stress and UPR were induced in HS-exposed animals [20,21,22,23,24]. It has been reported that chronic HS treatment induced splenic gene expression levels of UPR-related factors [21]. Ma et al. also showed that UPR activity was increased in skeletal muscle under chronic HS [22]. These investigations also suggested that ER stress could trigger apoptosis, and the attenuation of ER stress improved the adverse effects of HS in chickens. In addition, Miao et al. recently showed that acute and severe HS-exposed broiler chickens exhibited an increase in the hepatic gene expression levels of GRP78, GRP94, and XBP1 [23]. These results suggest that heat-induced ER stress may be a novel target when reducing heatstroke. In particular, HS affected the skeletal muscle and liver, which possibly has a significant impact on whole-body metabolism by altering fuel substrate dynamics [25,26,27]. Skeletal muscle under HS leads to oxidative damage, decreased glycogen and protein synthesis, and altered fatty acid metabolism [25,26,27,28]. The suppression of stress state in these peripheral tissues is also essential in mitigating the effects of HS; however, it has not yet been demonstrated whether the extent of the ER-folding capacity alleviates the adverse effects of acute and severe HS on the whole body and these peripheral tissues.

Based on previous studies, the present study focused on the effects of the dietary supplementation of 4-phenylbutyric acid (4-PBA), an alleviator of ER stress, on broiler chickens exposed to acute and severe HS conditions. 4-PBA is a low-molecular-weight fatty acid and non-toxic pharmacological compound that suppresses ER stress by directly reducing the amount of misfolded protein [29]. To examine the changes in peripheral tissues and metabolic changes in the whole body due to ER stress alleviation, this study investigated the effects of 4-PBA on ER stress response in skeletal muscle and liver, as well as the growth, cloacal temperature, and energy metabolism in broiler chickens.

## 2. Materials and Methods

### 2.1. Animal Experiment

**Figure 1:**
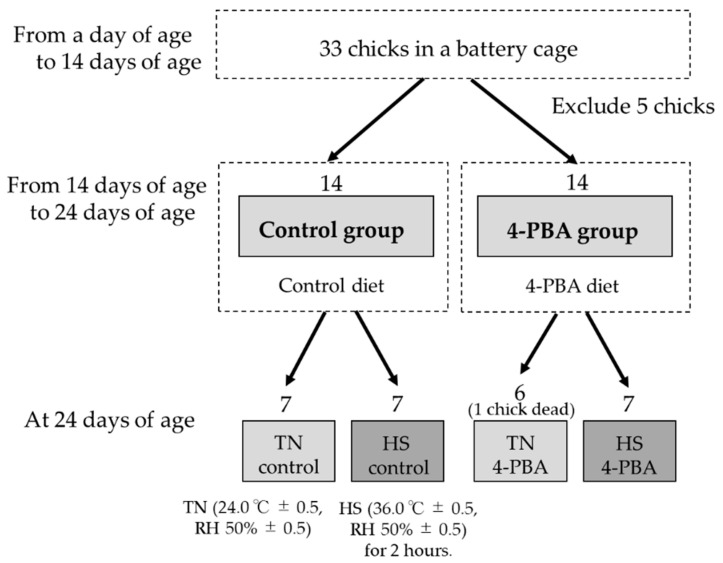
Animal experimental procedure. Twenty-eight male chicks were randomly allocated to two treatments (*n* = 14 for each treatment group) in a completely randomized design at 14 days of age. The chicks of the 4-PBA group were switched to the diet containing 4-PBA and fed for 10 days. At 24 days of age, the chicks were separated into 4 groups such that the mean weights of the groups were the same. Then, HS groups were exposed to HS (36.0 ± 0.5 °C, relative humidity 50.0 ± 5.0%) for 2 h. TN: thermoneutral group; HS: heat-stressed group; RH: relative humidity.

All the animal experiments were carried out according to the principles of the Basel Declaration, approved by the Tohoku University Institutional Animal Care and Use Committee, and performed under humane endpoints to minimize the pain of the broiler chickens. Thirty-three newly hatched male broiler chicks (Ross strain; *Gallus domesticus*) were purchased from a commercial hatchery (Katta-gun Zao, Miyagi, Japan). All the chicks were housed in an electrically heated battery cage. At 14 days of age, 5 chicks of markedly different weights were excluded from each group, and chicks were randomly allocated to two treatments with 14 chickens in a completely randomized design (Figure 1). All chickens were transferred into individual wire cages in environmentally controlled chambers and housed at an optimum temperature (24.0 °C ± 0.5, humidity 50% ± 0.5). The chickens were housed under 24 h light conditions throughout the experimental period. All chicks were fed the same experimental diet until 14 days of age. The composition of the experimental diet is presented in Table 1. 4-PBA (Tokyo Chemical Industry, Tokyo, Japan) was dissolved entirely in soybean oil and mixed with the same amount of feed as the control feed. The absolute 4-PBA content was set at 5.25 g per kg of diet feed to ensure that the average daily 4-PBA intake of the 4-PBA-fed group was >600 mg/kg body weight, according to previous reports using mice models [30,31,32]. At 14 days of age, chicks in the 4-PBA group were switched to the diet containing 4-PBA and fed for 10 days according to previous reports [33,34]. All the groups were allowed ad libitum access to feed and water throughout the experimental period. The feed intake and body weights were recorded daily.

### 2.2. Acute Heat Exposure

At 24 days old, chickens of two treatments were divided into the following 4 groups: (1) control under thermoneutral (TN control), (2) 4-PBA-supplemented under thermoneutral (TN 4-PBA), (3) control under HS (HS control), and (4) 4-PBA-supplemented under HS (HS 4-PBA) such that the mean weights of the groups were the same. At 24 days of age, two groups of HS treatments were exposed to a high temperature (36.0 ± 0.5 °C, relative humidity 50.0 ± 5.0%), and two groups of TN treatments were exposed to optimum temperature (24.0 ± 0.5 °C, relative humidity 50.0 ± 0.5%) conditions from 8:00 am to 10:00 pm, one time only (Figure 1). HS exposure was limited to two hours in order to minimize the risk of mortality. Cloacal temperature was simultaneously determined with a digital thermometer (±0.1 °C, Huger Electronics GmbH, Villingen-Schwenningen, Germany) before and after heat exposure. After two hours of heat exposure, blood was collected from all chicks and they were immediately euthanized by decapitation to obtain tissue samples (*n* = 7, 6, 7, 7; TN control, TN 4-PBA, HS control, HS 4-PBA group respectively). Isolated breast muscle and liver were frozen in liquid nitrogen, powdered, and stored at −80 °C until use.

### 2.3. Blood Analysis

Collected blood samples were immediately centrifuged at 1500× *g* for 15 min at 4 °C to separate plasma from blood cells. The blood plasma was collected in tubes as small aliquots and stored at −80 °C until analysis. Several blood analyses were performed using the following kits: glucose CII-Test to measure the glucose concentration, cholesterol E-test to measure the total cholesterol concentration, triglyceride E-test to measure the triglyceride concentration, and the NEFA-C-Test to measure the non-esterified fatty acid concentration (all from Wako Pure Chemical Industries, Osaka, Japan) according to the manufacturer’s instructions.

### 2.4. Quantitative RT-PCR

PCR analysis was performed as previously described [35]. In brief, total RNA was extracted from the isolated breast muscle and liver using TRIzol reagent (Thermo Fisher Scientific, Inc., Waltham, MA, USA) according to the manufacturer’s instructions. Total RNA was reverse-transcribed with mixed primers consisting of oligo (dT) and random hexamers into cDNA using M-MLV Reverse Transcriptase (28025013; Thermo Fisher Scientific, Inc.) according to the manufacturer’s instructions. Gene expression levels were determined using a TB Green^®^ Premix Ex Taq II Kit (RR820S; Takara Bio Inc., Shiga, Japan). The primer sequences are listed in Table 2. The results were normalized to the 18S rRNA level and shown as fold changes relative to the control value. PCR was performed using a CFX Connect™ system (Bio-Rad Laboratories, Hercules, CA, USA).

### 2.5. Statistical Analysis

All the data are presented as the mean ± standard error of the mean. All statistical analyses were performed using R version 4.0.3 (R Foundation for Statistical Computing, Vienna, Austria). All data were analyzed using a completely randomized design. To compare with the control and 4-PBA groups, the statistical significance of body weight (BW) at 24 days of age and feed conversion ratio (FCR) were analyzed using the Student’s *t*-test. Other results of statistical significance were determined using the 2-way analysis of variance (ANOVA), followed by a post hoc Tukey–Kramer test for comparisons between groups. *p* < 0.05 was considered to be significant.

## 3. Results

### 3.1. 4-PBA Supplementation in Diets Did Not Affect Growth, Food Intake, and Tissue Weight

At 24 days of age, broiler chickens were subjected to BW and FCR between 14 and 24 days old. No significant differences were observed between the control and 4-PBA groups (BW = 1010 ± 55.6 (g) vs. 1029 ± 37.7, *p* = 0.700); (FCR = 1.46 ± 0.0397 vs. 1.41 ± 0.0381, *p* = 0.301). After exposure to heat stress, neither the control nor the 4-PBA group showed changes in breast muscle or liver weight (Table 3). These results revealed that 4-PBA had no adverse effects on the growth performance of broiler chickens. In addition, plasma concentrations of the major liver function markers, aspartate aminotransferase and alanine aminotransferase were not different or lower in the 4-PBA groups compared with the control groups (Table 3). This indicates that 4-PBA supplementation had no toxicology for broiler liver.

### 3.2. 4-PBA Supplementation in Diets Attenuated Hyperthermia and Plasma Metabolites Changes in Heat-Stressed Broiler Chickens

**Figure 2:**
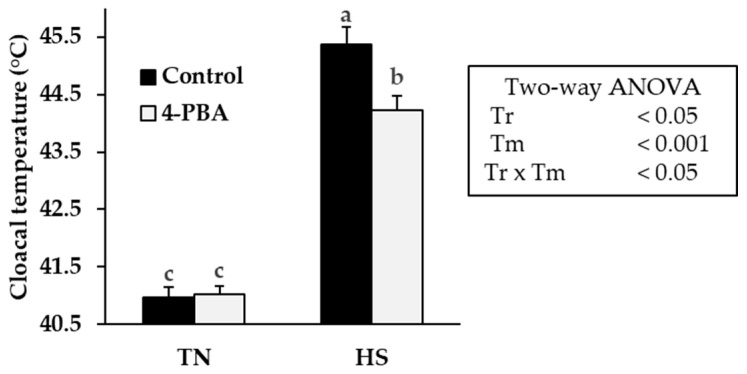
Effects of 4-PBA on the cloacal temperature in the heat-stressed broiler chickens. The cloacal temperature of broiler chickens fed control or 4-PBA supplementation in diet before and after heat exposure for 2 h. Data show means ± SE (*n* = 6–7). The presence of different letters indicates statistical significance. Tr: treatment; Tm: temperature.

Compared with conditions under optimal temperature (41.0 ± 0.18 °C for TN control group, and 41.0 ± 0.15 °C for TN 4-PBA group), cloacal temperature was drastically increased in both the control and 4-PBA-fed groups after two hours of acute HS (Figure 2). Notably, the elevation of body temperature was suppressed in the 4-PBA-fed group (44.2 ± 0.25 °C) compared to that of the control (45.4 ± 0.31 °C). In the thermoneutral (TN) group, there were no changes in the plasma metabolite levels between the control and 4-PBA groups (Table 4). Under HS, plasma glucose concentrations increased in chickens fed a 4-PBA-supplemented diet compared to those in the control group. In contrast, plasma NEFA concentrations were decreased in the 4-PBA supplemented group compared with those of the control group. Plasma cholesterol and triglyceride concentrations were not affected by heat or 4-PBA supplementation.

### 3.3. 4-PBA Supplementation in Diets Attenuated ER Stress of Skeletal Muscle but Not Liver

**Figure 3:**
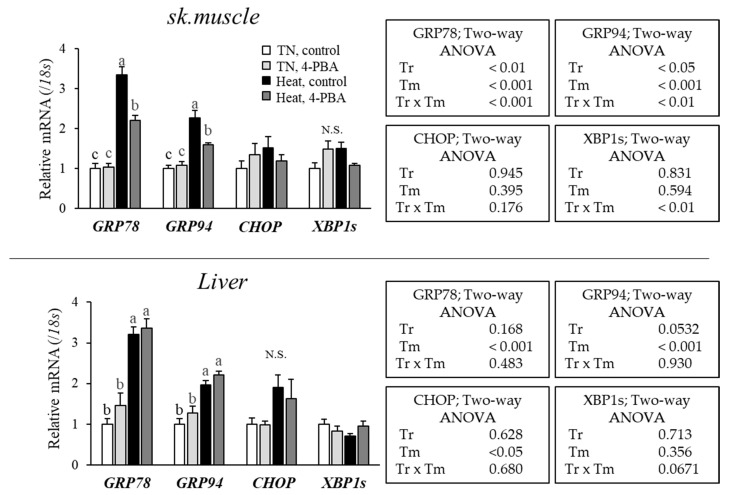
Effects of 4-PBA on UPR relative gene expression in the skeletal muscle and liver under acute HS. Relative mRNA expression of UPR relative genes (GRP78, GRP94, CHOP, and XBP1s) of thermoneutral (TN) and HS broiler chickens fed control or 4-PBA diet. Data show means + SE (*n* = 6–7). Different letters indicate statistical significance (*p* < 0.05). Tr: treatment; Tm: temperature.

The HS group showed increased GRP78 and GRP94 as markers of the initial response genes of UPR in skeletal muscle and liver (Figure 3). However, CHOP and XBP1s did not display significant changes in either tissue. 4-PBA significantly reduced GRP78 and GRP94 expression in skeletal muscle under HS compared to the control-diet-fed group. There were no changes in gene expression in the liver between the 4-PBA and control groups.

## 4. Discussion

In the present study, we fed broiler chickens a diet supplemented with 4-PBA, which acts as an ER stress inhibitor, and investigated its response to acute HS. Broiler chickens of 24 days of age were used because they no longer require constant heat incubation; conversely, they are affected by HS from this age. Surprisingly, our results suggest that 4-PBA significantly suppressed or delayed the heat-induced elevations in cloacal temperature. The acute exposure of broiler chickens to high ambient temperatures results in rapid increases in cloacal temperature, with maximum values reaching 46 °C [36]. 4-PBA decreased cloacal temperature by approximately 1 °C compared to the control, which may significantly impact the survival of the broiler chickens. A similar report showed that the inhibition of ER stress by 4-PBA extended the survival time of mice exposed to severe HS [37]. These data suggest that 4-PBA prevents the progression of heatstroke and may positively affect thermal homeostasis. However, it is unclear how 4-PBA is involved in thermoregulation.

In addition, we investigated whether 4-PBA affects energy metabolism. 4-PBA did not alter the concentrations of plasma metabolites under thermoneutral conditions. 4-PBA suppressed the elevation of plasma NEFA concentrations under acute HS. The plasma’s high NEFA and low glucose concentrations are characterized in chickens exposed to acute heat exposure [38,39]. The present results confirmed a decrease in plasma glucose and an increase in NEFA concentrations under HS conditions compared to the control group. This suggests that HS affects the physiology and energy metabolism levels of broiler chickens. In contrast, 4-PBA supplementation reduced the elevation of NEFA to about half of that in the control group and completely restored glucose depletion under HS conditions. Therefore, 4-PBA, acting as a suppresser of ER stress by directly reducing the amount of misfolded protein [29], maintained constant NEFA and glucose concentrations by suppressing energy consumption under acute HS.

Acute HS in broiler chickens increases the expression of GRP78 and GRP94, which are involved in the initial response of the UPR, which is mainly induced by ATF6 [11]. In contrast, XBP1 and CHOP expression did not change in either tissue. These genes were not induced because the heat exposure was as short as two hours and only the ATF6-downstream genes were activated. The present result is partially consistent with a previous report showing that the molecular chaperones GRP78, GRP94 and XBP1 genes were increased, but no increase in the PERK-ATF4-CHOP signaling was observed in chicken livers under acute HS (35 °C, 6 h) [21]. 4-PBA reduced the expression of heat-induced GRP78 and GRP94 in skeletal muscles but not in the liver. To the best of our knowledge, while this study is the first to focus on the effect of 4-PBA on alleviating ER stress in the liver of broiler chickens exposed to HS, there are several reports of 4-PBA reducing hepatic ER stress and alleviating hepatotoxicity in vivo [40,41,42,43]. These findings have established that 4-PBA also has an effect on ER stress attenuation in the liver. Thus, our current results suggest that 4-PBA was not effective in attenuating ER stress which is induced immediately after HS in the liver of the young broiler chicken, but further time- and dose-elapsed analysis under acute HS is needed. It should be considered that the liver finding in this study may be derived from using the young broilers, which may have different responses to heat and 4-PBA compared to older birds. Therefore, differences in day-old-dependent responses also need to be clarified. Moreover, recent studies reported that ER stress in the liver is caused by various factors, such as changes in redox imbalance and lipotoxicity [44,45]. Therefore, it is suggested that ER stress in the liver may be induced by a different mechanism to that in the skeletal muscle.

These results demonstrated that 4-PBA suppressed the elevated body temperature, plasma metabolite changes, and ER stress inhibition in the skeletal muscles of broiler chickens under acute HS. As fasting was shown to affect the tolerance to acute HS [46,47], 4-PBA might be involved in the metabolism and nutritional status under acute HS and contribute to the acquisition of heat tolerance. Further research is needed to elucidate the mechanisms that contribute to the heat tolerance of 4-PBA.

## 5. Conclusions

In conclusion, the current study clearly showed that the dietary supplementation of 4-PBA (5.25 g per kg of diet feed) repressed heat-induced hyperthermia and plasma NEFA elevation. Furthermore, 4-PBA decreased the expression of heat-induced GRP78 and GRP94 in the skeletal muscle of broiler chickens. As 4-PBA did not decrease acute HS-induced elevation of gene expression in the liver, it might be suggested that 4-PBA improved ER stress in a tissue-specific manner. Thus, the present study provides direct evidence that ER stress is involved in the regulation of heatstroke in young broiler chickens.

## Figures and Tables

**Table 1:** Composition of the experimental diet.

Ingredient	(%)
Corn	54.0
Soya bean meal	36.2
Soya bean oil	4.86
Salt	0.600
Limestone	1.00
Dicalcium phosphate	1.75
Glucose	0.670
Choline chloride	0.130
DL-methionine	0.250
L-lysine hydrochloride	0.040
Magnesium sulfate	0.300
Vitamin premix *^1^	0.100
Mineral premix *^2^	0.100
Total	100
Calculated Nutritional Values	
ME (Mcal/kg) *^3^	3.10
CP (%) *^4^	21.0
Methionine (g/kg)	5.50
Sulphur amino acids (g/kg)	8.96
Lysine (g/kg)	12.4
Threonine (g/kg)	7.97
Arginine (g/kg)	13.7
Tryptophan (g/kg)	2.52
Calcium (g/kg)	9.23
Available phosphorus (g/kg)	4.80

*^1^ Provided per kilogram of diet: retinol acetate, 1 mg; cholecalciferol, 5 µg; α-tocopherol acetate, 10 mg; thiamin hydrochloride, 1.8 mg; riboflavin, 3.6 mg; pyridoxine hydrochloride, 3.5 mg; calcium pantothenate, 10 mg; 2-methyl-1,4-naphthoquinone, 0.5 mg; folic acid, 0.55 mg; cyanocobalamin, 0.01 mg; biotin, 0.15 mg. *^2^ Provided per kilogram of diet: MnSO_4_·5H_2_O, 316.4 mg; ZnSO_4_, 129.5 mg; FeSO_4_·7H_2_O, 522 mg; CuSO_4_, 26.34 mg, KI, 0.6 mg; Na_2_SeO_3_, 3.92 mg; CoCl_2_·6H_2_O, 3.92 mg; MoO_3_, 0.6 mg. *^3^ ME = metabolizable energy. *^4^ CP = crude protein.

**Table 2:** Primer sequences.

Gene Name	Accession No.	Sequence (5′–3′)	Product Length
			(bp)
*GRP78*	NM_205491	Fwd: GAA TCG GCT AAC ACC AGA GGA	118
		Rev: CGC ATA GCT CTC CAG CTC ATT	
*GRP94*	NM_204289	Fwd: CAA AGA CAT GCT GAG GCG AGT	186
		Rev: TCC ACC TTT GCA TCC AGG TCA	
*CHOP*	HAEK01137550	Fwd: GAG GAC AAA GCG GAA GCG T	232
		Rev: GAA GCC ATC AGT CCA TGC CA	
*XBP1s*	NM_001006192	Fwd: CTA CGG ATG TGA AGG AAT CCC AGG	75
		Rev: CTG CAC CTG CTG CGG ACT CA	
*18* *S*	XR_005857224	Fwd: TAG ATA ACC TCG AGC CGA TCG	312
		Rev: GAC TTG CCC TCC AAT GGA TCC	

**Table 3:** Average body weight (g), feed conversion ratio (FCR), and tissue weight (g/kg BW).

	TN		HS			*p*-Value		
	Control	4-PBA	Control	4-PBA	SE	Tr	Tm	Tr ×Tm
Tissue weight (g/kg BW)								
Breast muscle *^1^	106 ^a^	102 ^a^	99.1 ^b^	91.7 ^b^	2.09	0.124	0.0267	0.725
Liver	25.0	21.9	22.6	22.3	0.587	0.186	0.280	0.251
Liver function marker (U/L)								
Aspartate aminotransferase *^2^	275	207	250	226	9.97	0.167	0.755	<0.05
Alanine aminotransferase	15.1	13.7	15.1	14.6	0.566	0.432	0.710	0.705

Broiler chickens were fed either the control or 4-PBA diet (*n* = 6–7). Different letters indicate statistical significance (*p* < 0.05). TN: thermoneutral group; HS: heat-stressed group; Tr: treatment; Tm: temperature. *^1^ pectoralis major weight. *^2^ not significant by the Tukey–Kramer post hoc test.

**Table 4:** Blood parameters of broiler chickens.

Parameters	TN		HS			*p*-Value		
	Control	4-PBA	Control	4-PBA	SE	Tr	Tm	Tr × Tm
Glucose (mmol/L)	15.7 ^ab^	16.4 ^ab^	13.1 ^b^	17.8 ^a^	0.557	<0.01	0.383	<0.05
Cholesterol (mg/L)	1226	1195	1411	1206	42.9	0.160	0.261	0.309
Triglyceride (mmol/L)	0.410	0.373	0.372	0.287	0.0243	0.216	0.200	0.623
NEFA (mEq/L)	0.197 ^b^	0.149 ^b^	0.584 ^a^	0.338 ^ab^	0.0507	<0.05	<0.01	0.236

Data show means ± SE (*n* = 6–7). Different letters indicate statistical significance (*p* < 0.05). TN: thermoneutral group; HS: heat-stressed group; Tr: treatment; Tm: temperature.

## Data Availability

The data presented in this study are available on request from the corresponding author.
